# Effects of Two Doses of Curry Prepared with Mixed Spices on Postprandial Ghrelin and Subjective Appetite Responses—A Randomized Controlled Crossover Trial

**DOI:** 10.3390/foods7040047

**Published:** 2018-03-26

**Authors:** Sumanto Haldar, Joseph Lim, Siok Ching Chia, Shalini Ponnalagu, Christiani Jeyakumar Henry

**Affiliations:** 1 Clinical Nutrition Research Centre (CNRC), Singapore Institute for Clinical Sciences (SICS), Agency for Science Technology and Research (A*STAR), 30 Medical Drive, Singapore 117609, Singapore; sumanto_haldar@sics.a-star.edu.sg (S.H.); joseph_lim@sics.a-star.edu.sg (J.L.); smileychiasc@gmail.com (S.C.C.); Shalini_Ponnalagu@sics.a-star.edu.sg (S.P.); 2 Department of Biochemistry, National University of Singapore, Singapore 119077, Singapore

**Keywords:** spices, curry, ghrelin, appetite response

## Abstract

Spices are known to provide orosensory stimulation that can potentially influence palatability, appetite, and energy balance. Previous studies with individual spices have shown divergent effects on appetite and energy intake measures. In a real-life context, however, several spices are consumed in combinations, as in various forms of curries. Therefore, we investigated changes in postprandial appetite and plasma ghrelin in response to the intake of two doses of curry prepared with mixed spices. The study was undertaken in healthy Chinese men, between 21 and 40 years of age and body mass index ≤27.5 kg/m^2^. Appetite was measured using visual analogue scales (VAS) and plasma ghrelin was measured using multiplex assay. Compared with the control meal (Dose 0 Control (D0C), 0 g mixed spices), we found significantly greater suppression in ‘hunger’ (both *p* < 0.05, after Bonferroni adjustments) as well in ‘desire to eat’ (both *p* < 0.01) during the Dose 1 Curry (D1C, 6 g mixed spices) and Dose 2 Curry (D2C, 12 g mixed spices) intake. There were no differences, however, in plasma ghrelin or in other appetite measures such as in ‘fullness’ or in ‘prospective eating’ scores. Overall, the results of our study indicate greater inter-meal satiety due to mixed spices consumption, independent of any changes in plasma ghrelin response.

## 1. Introduction

Spices and related flavor compounds consumed worldwide are known to provide orosensory stimulation which can potentially influence palatability and appetite. This in turn can modulate ingestive behavior within meals and between meals and thereby have the potential to alter energy balance [1]. It is an established fact that taste and smell can influence satiety and hunger responses [2,3], although the associations can be divergent, depending on the individual food types [4], as well as on the optimal dosing of sensory intensity [5]. The literature on the intake of individual spices *per se* indicate that the associations between sensory, appetite, and energy intake to be rather equivocal, with some studies showing an increased palatability/liking of foods when spices are added to them [6], whereas other studies finding no such differences [7]. Similarly, regarding appetite response, the findings have been rather variable, with some studies showing no differences in appetite ratings when pepper, ginger, horseradish, etc. were individually added to a mixed dish [8],whereas a recent animal study found appetite enhancing effects of essential oils from both cinnamon and ginger [9]. Similarly, increased use of spices have also been reported in individuals with compromised chemosensory function in order to compensate for the loss of appetite [10], supporting the appetite modulating ability of spices.

In the recently completed ‘Polyspice study’ we found significant improvements in postprandial glucose homeostasis as well as increases in glucagon like peptide- (GLP-1) response [11,12]. Given the equivocal nature of findings in the current literature regarding spice intake and appetite response, we further explored whether the subjective appetite response will differ between two doses of curry, made with polyphenol rich mixed spices, in comparison with non-curry control within the same Polyspice study cohort. Furthermore, while it is reasonably well established that postprandial ghrelin response can differ depending on the macronutrient composition of the meals [13,14], less is known regarding the effects of dietary bioactive phytochemicals on ghrelin response. Evidence is beginning to emerge that polyphenol content of foods may influence postprandial ghrelin response [15,16,17], with different polyphenols suggested to have divergent effects [18]. Therefore, we additionally investigated the postprandial ghrelin response to two different doses of curry made with polyphenol rich mixed spices and vegetables. To the best of our knowledge, this is the first dose-response study of its kind exploring the effects of dietary relevant doses of curry intake, made with mixed spices, on postprandial appetite and ghrelin response.

## 2. Materials and Methods

The details of the study design have been described previously [11]. In brief, the study was undertaken in healthy Chinese men between 21 and 40 years of age and body mass index ≤27.5 kg/m^2^. The study was approved by the Domain Specific Research Board (DSRB) ethics committee, Singapore (Reference: C/2015/00729) and was registered on clinicaltrials.gov (Identifier No. NCT02599272). This was a 3-way randomized crossover trial, with each volunteer completing three separate study sessions: Dose 0 Control (D0C), or Dose 1 Curry (D1C), or Dose 2 Curry (D2C) treatments. Twenty volunteers completed D0C and D2C sessions, whereas 17 volunteers completed the D1C (optional) session. During the individual study sessions, each volunteer consumed the test meals for breakfast, after an overnight fast following a standardized dinner the evening before. D0C meal contained no (0 g) spices, D1C meal consisted 6 g of mixed spices, and D2C meal consisted of 12 g mixed spices. The mixed spices preparations for D1C and D2C were identical and were prepared by thoroughly mixing dried powders of different spices consisting of turmeric, coriander seeds, cumin seeds (all Everest Spices, Mumbai, India), dried Indian gooseberry (‘*amla*’, *emblica officinalis*, Ramdev Spices, Ahmedabad, India), cayenne pepper (Robertson’s, Durban, South Africa), cinnamon (McCormick’s, Baltimore, MD, USA), and clove (Robertson’s, Durban, South Africa) and were mixed in the ratio of 8:4:4:4:2:1:1, respectively. Test meals were consumed with a portion of white rice, providing a total of approximately 100 g available carbohydrates each and were balanced for total energy, protein, fat, dietary fiber, and total vegetables content. The mean total energy contents for each test meal was approximately 605 kilocalories consisting around 67%, 7%, and 27% energy from carbohydrate, protein, and fat, respectively. Volunteers were asked to consume their meals within 15 min of serving.

Visual analogue scales (VAS) were used to rate subjective appetite sensations as validated previously [19,20]. The VAS consisted of 100 mm long horizontal lines with two ends describing “extremely…” (coded as 100 mm) and “not at all…” (coded as 0 mm) with each scale measuring ‘hunger’, ‘fullness’, ‘desire to eat’, and ‘prospective eating’. Volunteers were asked to capture their appetite sensations during various times, at regular intervals, by putting vertical mark along the four horizontal lines. These measurements were undertaken immediately prior to the consumption of test meals (baseline, 0 h) followed by 7 postprandial time points at various regular intervals (0.25 h, 0.5 h, 1.0 h, 1.5 h, 2.0 h, 2.5 h, 3 h). Blood samples were also obtained for plasma ghrelin measurements via an intravenous cannula collected during the same times as the appetite response measurements (except for 0.25 h time point). Blood samples were collected in 2 mL K_2_ Ethylenediaminetetraacetic acid (EDTA) vacutainer tube (BD, Franklin Lakes, NJ, USA) pre-treated with cOmplete™, Mini, EDTA-free protease inhibitor cocktail tablet (Roche, Basel, Switzerland), stored cool on ice, and were centrifuged within 45 min of collection at 1500× *g* for 10 min at 4 °C. Plasma samples were then immediately stored at −80 °C until analyses. Luminex^®^ bead-based multiplex assays, based on Luminex^®^ xMAP^®^ technology, were used to measure total ghrelin concentration in plasma according to manufacturer’s protocol (ProcartaPlex, Thermo Fisher Scientific, Waltham, MA, USA).

Statistical analysis was performed using SPSS, version 24 (IBM Inc., Armonk, North Castle, NY, USA). Data were analyzed using the mixed effects model with the doses as the fixed effect using a compound symmetry covariance structure to test for the overall effect of the doses. Change from baseline response for plasma ghrelin and appetite measures were calculated as the change from baseline areas under the curve (ΔAUC). Post-hoc pairwise comparisons, using Bonferroni corrections, were used to compare differences in change from baseline AUCs between various doses. Furthermore, to test the overall effect of the doses on total AUC data for plasma ghrelin, the corresponding baseline values (fasting values) of the subjects at each of the doses were added as a covariate, with the doses as the fixed effects using a compound symmetry covariance structure. However, for the overall tests of the change from baseline AUCs, no covariates were used. Square root transformations of the data were undertaken to achieve normal distribution, where necessary.

## 3. Results

**Figure 1:**
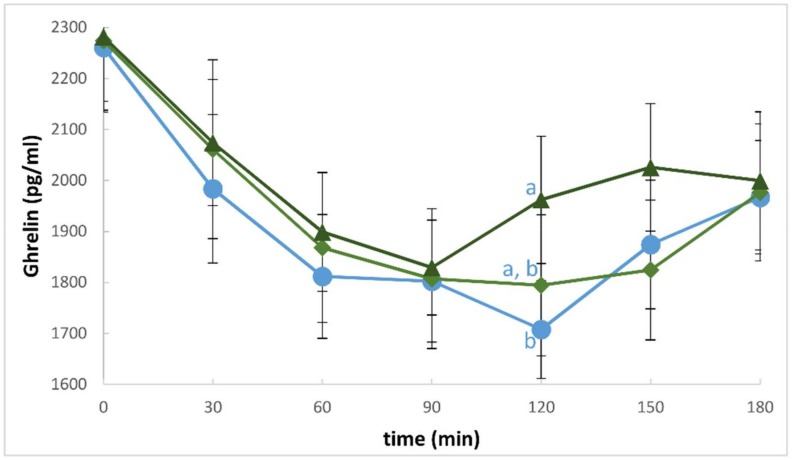
Mean (±Standard Error of Mean) postprandial plasma ghrelin concentration in response to the intake of three test meals: ● Dose 0 Control (D0C), ♦ Dose 1 Curry (D1C), ▲ Dose 2 Curry (D2C). The differences between the treatments at each time point were measured after controlling for the baseline values. Treatments that were significantly different from each other (*p*-value < 0.05) are represented by different letters. At time 120 min, there was a significant main effect of the treatment, although there was only marginal significance observed between Dose 0 and Dose 2 (*p*-value = 0.056), as shown in the figure above.

**Figure 2:**
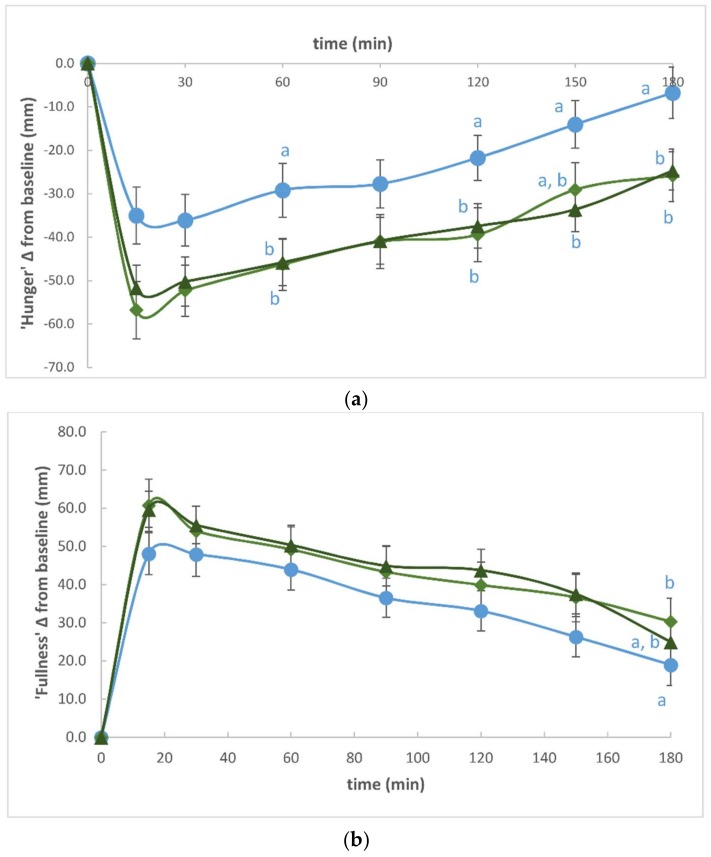

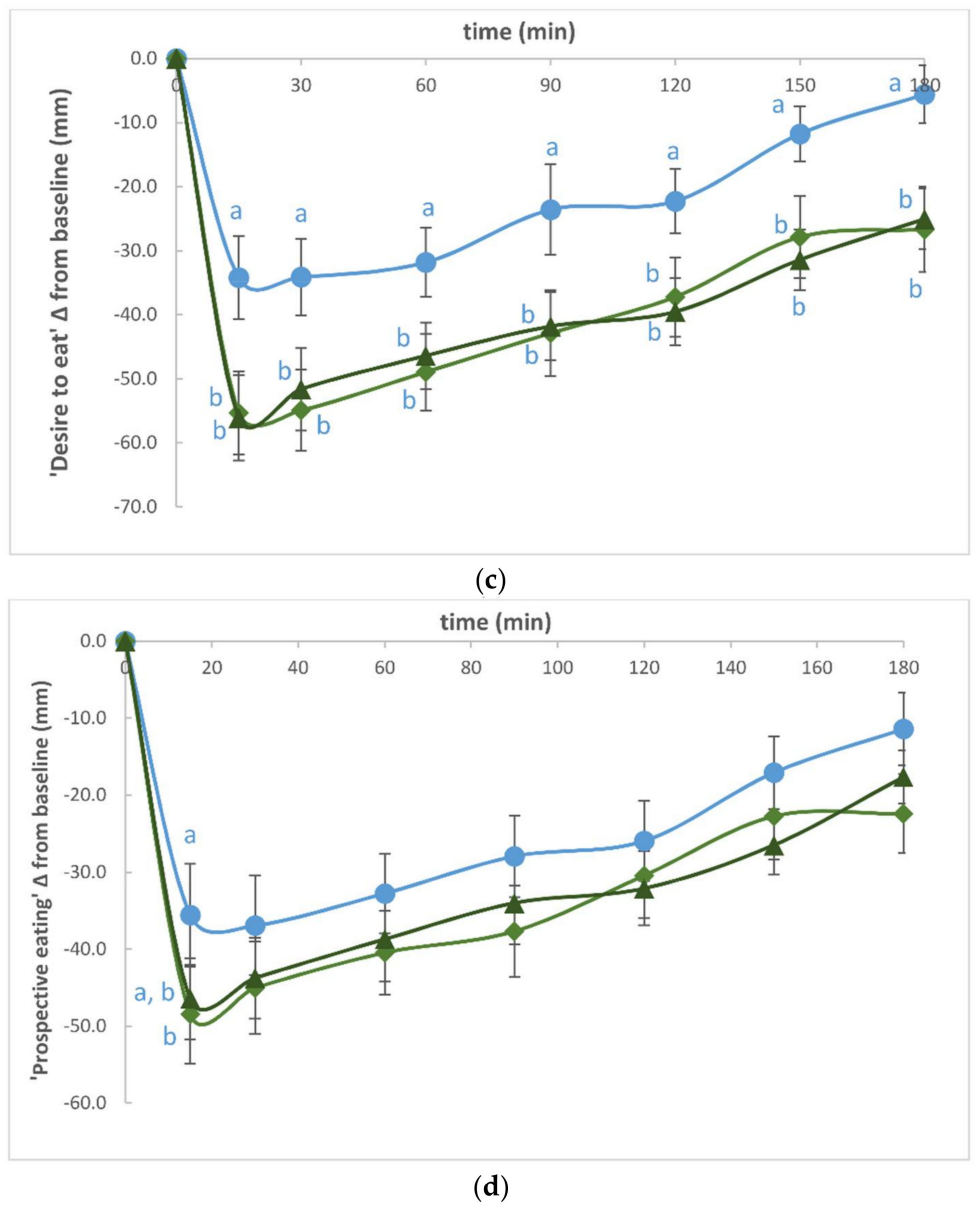
Mean (±SEM) in subjective appetite ratings over a 3 h period following consumption of three test meals. (**a**) ‘Hunger’; (**b**) ‘Fullness’; (**c**) ‘Desire to eat’; (**d**) ‘Prospective eating’. ● Dose 0 Control (D0C), ♦ Dose 1 Curry (D1C), ▲ Dose 2 Curry (D2C). In all the appetite ratings, the main effect of time and treatment were significantly different (*p*-value < 0.05). Treatments that were significantly different from each other (*p*-value < 0.05) are represented by different letters at each given time point.

There were no reported adverse reactions to the test meals, and the volunteers consumed their served meal portions in full within the suggested time allocated, indicating a satisfactory tolerance. Postprandial ghrelin response to the three test meals is shown in Figure 1. There were no significant differences in either the change from baseline AUC (∆AUC) or in the total areas under the curve (total AUC) between the D0C, D1C, and D2C meals. The mean change from baseline (0 h) of subjective appetite ratings including ‘hunger’, ‘fullness’, ‘desire to eat’, and ‘prospective eating’ are shown in Figure 2. Compared with the control (D0C) test meal, D1C and D2C led to significantly greater suppressions in ‘hunger’ (by approximately 54% and 51% during D1C and D2C, respectively, as compared with D0C control meal, both *p* < 0.05, after Bonferroni corrections) as well as in the ‘desire to eat’ (by approximately 62% and 60% in D1C and D2C, respectively, as compared with D0C control meal, both *p* < 0.01, after Bonferroni corrections), as calculated using the areas under the curve of the change from baseline measurements. The summary data are shown in Table 1. Moreover, even though the mean change from baseline AUC (∆AUC) of postprandial ‘fullness’ was also greater by about 20% during D1C and D2C meals as compared with the D0C meal, indicating increased ‘fullness’, none of the differences reached statistical significance. Similarly, there were no statistical differences in the ‘prospective eating’ rating between test meals.

## 4. Discussion

It is generally believed that glucose homeostasis is associated with appetite and/or incretin hormone response and vice-versa [21,22,23]. While several studies have investigated these effects simultaneously by modulating macronutrient compositions of meals, a limited number of studies have explored the effects of mixed spices in humans using a controlled dose-response trial [1]. Given that we have previously shown beneficial effects of curry made with mixed spices on glucose homeostasis [11], in this additional investigation, we wanted to explore the influence of dose-dependent increases in the intake of curry made with mixed spices on appetite and ghrelin responses. The strengths of our study design were the use of dietary relevant doses of mixed spices that are typically consumed in Indian curries and that the study was deliberately undertaken in a Chinese population, who would not usually consume large amounts of Indian curries, thereby avoiding any potential residual effects due to habituation. Moreover, we also balanced the total energy, macronutrients, and total vegetable contents across the three test meals.

Despite all the test meals being well tolerated, we found significant increases in ‘hunger’ suppression and in the suppression of ‘desire to eat’ with the mixed spice containing curry doses as compared with the control meal. This indicates that the consumption of spices may increase inter-meal satiety, which ties-in with our previously reported finding within the same study of an increase in plasma GLP-1 concentration with increasing curry doses [12], which can partly lead to appetite suppression. Other studies have also shown that consumption of individual spices can lead to increases in plasma GLP-1 [24] as well as in peptide YY (PYY) [25] concentrations. Furthermore, the lack of any difference in postprandial ghrelin response between the various test meals in our study is similar to the observation made with two doses of cinnamon added to 300 g of rice pudding [24]. The consistencies in these findings may suggest that the appetite suppression effects of spices may be via the increases in the in vivo concentrations of anorexigenic gut hormones such as GLP-1 and PYY, rather than via an increased suppression of the orexigenic hormone ghrelin. In further support of our findings, a recent study also found appetite suppressing effects of individual spices (e.g., turmeric, cinnamon, ginger) [25]. All these spices (and more) were used together in the mixed spice containing test meals in our study. In contrast to our findings, however, another study investigating the effects of individual spices such as mustard, horseradish, black pepper, and ginger found negligible effects on appetite response [8]. Therefore, different spices are likely to give rise to divergent effects. As such, spices such as chili have been shown to demonstrate appetite stimulating effects [26]. The ‘appetizer effects’ of spices have also been previously shown by adding them in low salt-foods [27] as well as to reduced fat foods [6]. The contrasting effects of different spices on appetite could be resulting from the diverse ways by which individual spices can affect the sensory and molecular pathways, as have been reviewed in detail elsewhere [1].

Within our study population we found a modest inter-individual variability in the subjective appetite ratings as a result of mixed spice consumption at the various doses, as observed through the large standard deviations in appetite rating measures (Table 1). This could partly be due to differences in prior familiarities and likings, since it has been shown in a recent study that familiarity of spices can determine pleasantness response which in turn can affect appetite [28]. Indeed, prior preference for an individual to sweet or savory foods has been shown to determine the quantity of either sweet or savory foods that is eaten in an experimental setting [29]. There are also indications that behavioral variables including risk taking and different personality traits could also influence the motivation to consume spicy foods [30], and although this previous study was undertaken within the Western dietary context, we used Chinese males who may also have different affiliations towards eating Indian curries. In our study, however, we did not gather any information on prior familiarity, liking, or on personally traits which could directly or indirectly explain part of the inter-individual variabilities in appetite responses.

We did not find any obvious dose-response associations with appetite measures in our study, since both the curry doses seemed to have exerted similar effects as compared with the non-curry control meal. This was despite previous studies suggesting an inverted U-shaped (Wundt) curve between sensory intensity and appetite response as discussed in more detail by McCrickerd et al. [5]. More specifically, both palatability [31] as well as appetite in response to dose-dependent increases in flavor/taste intensities observed this inverted U-shaped phenomenon by several investigators [32,33], although we did not find such effects. These differences in findings could be explained by the fact that the “preferred” taste intensity for spices could have varied between individuals, which may have contributed to the moderate inter-individual variability in appetite response seen in our study. This in turn could lead to lack of any obvious dose-response effects. Moreover, we measured neither the volunteers’ innate taste preference nor the palatability of the test meals, which were some of the limitations of this study. Additionally, the volunteers were required to consume the entire amount of test meals served, irrespective of their within meal satiation. Furthermore, the study could have been more objective if we provided them an *ad libitum* meal for lunch and measured the volunteers’ actual food intake rather than solely measuring subjective appetite response between meals. Since the primary objective of our study was to investigate metabolic response to standardized, fixed amounts of foods, the study of *ad libitum* food intake measurement was not an option. Finally, in addition to taste, the aroma from the release of volatile compounds from spices could have also contributed to greater appetite suppression. It is well recognized that aroma from foods and non-food materials reaching the olfactory epithelium via the orthonasal and the retronasal routes can lead to appetite suppression [34,35].

## 5. Conclusions

The results from our study indicate that mixed spices consumption can lead to greater increases in inter-meal satiety through suppression in both ‘hunger’ as well as in ‘desire to eat’ in the period immediately subsequent to the meal. Both differences in taste intensities as well as aroma from the addition of mixed spices may have contributed to a greater satiety value of the mixed spice containing meals. These changes in postprandial appetite responses seem to be independent of changes in plasma ghrelin concentration, although could potentially have been related to the increase in postprandial plasma GLP-1 concentration, as found within the same cohort previously. The postprandial effects of individual spices or mixed spices on other appetite hormones remain to be established. It should be noted that this was a single-meal, acute feeding study and any long-term effects remain to be further investigated.

## Figures and Tables

**Table 1:** Postprandial changes from baseline (ΔAUC) over 3 h periods in plasma ghrelin concentration and appetite response measures during three test meals (D0C, D1C, and D2C).

Measurement	D0C (Mean ± SD)(*n* = 20)	D1C (Mean ± SD)(*n* = 17)	D2C (Mean ± SD)(*n* = 20)	Pairwise Comparison *
Total Ghrelin (ΔAUC)	−78,098.98 ± 41,101.98	−76,549.46 ± 41,482.70	−78,883.94 ± 40,022.88	ND
‘Hunger’ (ΔAUC)	−4659.45 ± 3272.36	−7179.61 ± 3782.34	−7020.96 ± 3871.20	D0C vs. D1C (*p* = 0.017)
				D0C vs. D2C (*p* = 0.028)
‘Fullness’ (ΔAUC)	6393.71 ± 3681.80	7764.54 ± 3908.07	7850.15 ± 3581.19	ND
‘Desire to Eat’(ΔAUC)	−4495.00 ± 3194.31	−7268.09 ± 4053.52	−7194.88 ± 3849.50	D0C vs. D1C (*p* = 0.002)
				D0C vs. D2C (*p* = 0.005)
‘Prospective eating’ (ΔAUC)	−4913.78 ± 3401.05	−6095.76 ± 3612.99	−5883.42 ± 3506.46	ND

* Pairwise comparisons after Bonferroni correction. Δ AUC—changes from baseline area under the curve, ND—no significant difference, D0C—Dose 0 Control, D1C—Dose 1 Curry, D2C—Dose 2 Curry (D2C—), SD—standard deviation.
